# Pre-concentration by liquid intake by paper (P-CLIP): a new technique for large volumes and digital microfluidics†

**DOI:** 10.1039/c7lc00440k

**Published:** 2017-06-12

**Authors:** Darius Rackus, Richard P. S. de Campos, Calvin Chan, Maria M. Karcz, Brendon Seale, Tanya Narahari, Christopher Dixon, M. Dean Chamberlainab, Aaron R. Wheeler

**Affiliations:** 1 ^a^Department of Chemistry, University of Toronto, 80 St George St., Toronto, ON, M5S 3H6, Canada; 2 ^b^Donnelly Centre for Cellular and Biomolecular Research, 160 College St., Toronto, ON, M5S 3E1, Canada; 3 ^c^Institute of Biomaterials and Biomedical Engineering, University of Toronto, 164 College St., Toronto, ON, M5S 3G9, Canada

## Abstract

Microfluidic platforms are an attractive option for incorporating complex fluid handling into low-cost and rapid diagnostic tests. A persistent challenge for microfluidics, however, is the mismatch in the “world-to-chip” interface – it is challenging to detect analytes present at low concentrations in systems that can only handle small volumes of sample. Here we describe a new technique termed pre-concentration by liquid intake by paper (P-CLIP) that addresses this mismatch, allowing digital microfluidics to interface withvolumes on the order of hundreds of microliters. In P-CLIP, a virtual microchannel is generated to pass a large volume through the device; analytes captured on magnetic particles can be isolated and then resuspended into smaller volumes for further processing and analysis. We characterize this method and demonstrate its utility with an immunoassay for *Plasmodiumfalciparum* lactate dehydrogenase, a malaria biomarker, and propose that the P-CLIPstrategy may be useful for a wide range of applications that are currently limited by low-abundance analytes.

## Introduction

Microfluidic devices for bioanalysis offer the advantages of small samples, low reagent consumption, automation, and portability. However, small sample volumes can prove a disadvantage for applications that require the detection of a target analyte present in low concentration. This challenge is typically solved by using a detector that is inherently very sensitive (*e.g*., fluorescence^1^ or mass spectrometry^2^) or by incorporating a molecular amplification strategy (e.g., gold nanoparticle mediated silver reduction^3,4^ or enzyme-linked amplification^5^). These strategies are quite useful, but they often increase the instrumental or methodological complexity of the assay, which can present different challenges for portability and analysis in the field. An alternative to these strategies is to incorporate an analyte-pre-concentration step into the analytical process.

A variety of methods have been developed for pre-concentration in microfluidics, relying on a wide range of physical phenomena. Broadly, these methods can be categorized as being dynamic or static.^6^ Dynamic methods concentrate analytes on the basis of electrokinetic equilibrium; examples include field amplified sample stacking,^7^ isoelectric focusing,^8^ and isotachophoresis.^9^ Static pre-concentration methods, on the other hand, work to accumulate the target analyte in a particular location through binding,^10^ filtration, ^11^ or solvent extraction.^12^ One static pre-concentration strategy that is particularly powerful is surface binding on magnetic-microparticles.^13^ In this technique, functionalized magnetic microparticles are used to capture target analytes from solution, and magnetic fields applied *via* integrated^14^ or external^15^ magnets are used to position the particles for sorting^16^ or to retain them while the bulk solution is exchanged. ^17,18^ Captured analyte can then be eluted from the particles for downstream analysis^19^ or analysis may occur *in situ* with particle-bound analytes and reporters.^20^

While surface-binding on magnetic microparticles can be a versatile pre-concentration technique, a disadvantage is the risk of clogging when used with microchannel systems.^21^ “Open”-format microfluidic systems such as those powered by digital microfluidics (DMF) eliminate the problem of clogging and have proven particularly useful for handling magnetic micro-particles^22^ for the analysis of small molecules, ^23,24^ proteins,^25–31^ and nucleic acids.^32,33^ In the most common DMF device format, discrete droplets of liquid are sandwiched between two plates: the bottom plate comprises an array of electrodes that is covered by an insulating dielectric layer and a hydrophobic layer, and the top plate comprises a ground electrode that is covered with a hydrophobic layer. Droplets are moved by sequential application of voltages to electrodes adjacent to the droplet. In this two-plate format, droplets cannot be manipulated in the *z*-axis but are unrestricted by any walls or barriers in the *xy*-plane.^34^

Despite its proven utility in handling magnetic microparticles, ^22–33^ DMF is limited in its ability to effect pre-concentrations of significant magnitude. Specifically, the difference between the smallest and largest volumes that can be (practically) manipulated on most DMF devices is often quite small – *e.g*., in the devices used here, this range runs from ∼0.9 μL (a “unit” droplet covering one electrode) to ∼9 μL (a droplet covering 10 electrodes; volumes larger than this are impractical to use). To overcome this limitation, sample pre-concentration for DMF can be performed by mating the technique to external instrumentation. For example, Jebrail *et al*.^33^ demonstrated a system in which RNA is extracted on functionalized particles connected *via* wide-bore tubing to a peristaltic pump. Up to 100 μL of blood can be pumped through the tubing, after which particles can be collected on the DMF device. This is a useful advance, but for many applications, it would be advantageous to integrate the pre-concentration functionality directly into the device, without requiring external instrumentation.

To address the challenge described above, we have developed a fully integrated digital microfluidic pre-concentration technique that we call “pre-concentration by liquid intakeby paper” (P-CLIP). We recently introduced one variant of P-CLIP *via* a proof-of-concept demonstration of its utility for immunoprecipitating proteomic samples prior to elution, collection and analysis off-chip by mass spectrometry.27 Here, we comprehensively characterize the performance of P-CLIP implemented in several different formats, and apply it to an on-chip immunoassay (with chemiluminescent detection) for *Plasmodium falciparum* L-lactate dehydrogenase (*Pf*LDH), a biomarker for malaria.^35^ These results suggest that P-CLIP may be useful for integrating sample pre-concentration for diagnostic assays for infectious disease. More generally, the new technique should be incorporated in any DMF assay relying on functionalized magnetic microparticles.^22–33^

## methods

### Reagents and materials

Unless otherwise specified, reagents were purchased from Sigma-Aldrich (Oakville, ON), and deionized water (DI water) with a resistivity of >18 MΩ cm was used to prepare all aqueous solutions. SureWick G028 glass fiber conjugate pad material was generously donated by EMD Millipore (Etobicoke, ON). All solutions used in DMF devices contained 0.1% (w/v) Tetronic 90R4 (BASF Corp., Germany) surfactant unless otherwise specified. Magnetic particle suspensions were used at the manufacturer's supplied density, unless otherwise specified.

Dynabeads® M-270 Streptavidin-coated paramagnetic particles (2.8 μm dia.) were modified with anti-*Pf*LDH monoclonal capture antibody (HCA158, clone number 14008, Bio-Rad, Oxford, UK) as follows. Capture antibody was purified and concentrated using Amicon® Ultra 0.5 mL centrifugal filters, MW cut-off 100 kDa (Millipore) according to the manufacturer's instructions into Dulbecco's phosphate buffered saline (PBS). Concentrated antibody was conjugated to biotin using a biotin (type B) conjugation kit (AbCam, San Francisco, CA) according to the manufacturer's instructions, and then stored at 4 °C until use. The particles in a 100 μL aliquot were immobilized with a magnet, the supernatant was removed, and the particles were washed four times by resuspending them in 100 μL aliquots of PBS with 0.05% Tween-20, immobilizing with a magnet, and removing the supernatant. A 100 μL aliquot of 1.25 μg mL^−1^ solution of biotinylated capture antibody was added to the particles, and the suspension was incubated for 30 min at room temperature with rotation. The particles were washed three times with PBS with 0.05% Tween-20 (as above), and then resuspended in Superblock™ Tris-buffered saline (TBS) blocking buffer (Thermo Fisher Scientific, Rockford, IL) containing 0.1% (w/v) Tetronic 90R4 to the same density as the stock particle suspension (6.7 × 10^8^ particles per mL).

The detection antibody, *Pf*LDH-specific monoclonal IgG (MBS313354, clone number B2127M; MyBioSource.com, San Diego, CA), was conjugated to horseradish peroxidase (HRP) as follows. Detection antibody was purified and concentrated using Amicon® Ultra 0.5 mL centrifugal filters, MW cut-off 100 kDa per the manufacturer's instructions into PBS. Concentrated antibody was conjugated to HRP using an AbCam HRP conjugation kit per the manufacturer's instructions.

Other DMF ELISA reagents included wash buffer, sample diluent, blocking buffer, conjugate solution, and chemiluminescent substrate, and were prepared *via* modifications from previously reported methods.^28^ Wash buffer was PBS supplemented with 0.1% (w/v) Tetronic 90R4. Sample diluent was PBS supplemented with 4% (w/v) bovine serum albumin (BSA) and 0.1% (w/v) Tetronic 90R4. Blocking buffer was Superblock^™^ TBS with 0.1% Tetronic 90R4. Conjugate solution was HRP-conjugated detection antibody (0.37 ng mL^−1^) in blocking buffer. Stable peroxide (H_2_O_2_) and luminol/enhancer solutions were adapted from SuperSignal^™^ ELISA Femto (Thermo Fisher Scientific, Rockford, IL) kits, each supplemented with 0.05% (w/v) Tetronic 90R4.

### Device fabrication and operation

Digital microfluidic devices comprised two plates and were fabricated using two different designs. Bottom plates were formed from Cr-coated glass (as detailed elsewhere^30^) at the University of Toronto Nanofabrication Centre (TNFC) or by inkjet printing conductive ink (as detailed elsewhere^36^). Bottom plates of devices fabricated from Cr-coated glass comprised a 15 × 4 array of square driving electrodes (2.2 mm × 2.2 mm each), 12 large reservoir electrodes (16.4 mm × 6.7 mm) and 8 dispensing electrodes (2.2 mm × 4.4 mm). Bottom plates of devices fabricated by inkjet printing comprised 92 roughly square interdigitated electrodes (2.8 mm × 2.8 mm), 10 reservoir electrodes (10 mm × 6.7 mm) and 10 dispensing electrodes (5.2 mm × 2.4 mm). Both Cr-on-glass and inkjet-printed bottom plates were coated with a layer of parylene C (∼7 μm thick) and a spin-coated layer of either Teflon-AF (DuPont, Wilmington, DE) or FluoroPel PFC 1101V (Cytonix, LLC, Bellville, MD) (each ∼70 nm thick). Top plates were fabricated by spin-coating Teflon-AF in FC-40 onto indium– tin oxide (ITO) coated glass slides (25 m × 75 mm) (Delta Technologies, Loveland, CO). Two layers of doublesided tape (3M Company, Maplewood, MN) were used as spacers (∼180 μm) between top and bottom plates. The volume of a single unit droplet (defined as a droplet that covers one driving electrode) was 900 nL or 1.2 μL on Cr-coated glass and inkjet-printed devices, respectively.

Digital microfluidic devices were interfaced *via* pogo-pin connectors to the open-source DropBot control system (http://microfluidics.utoronto.ca/dropbot/) and droplet movement (driven by applying voltages of 85–110 V_RMS_ at 10 kHz) was programmed by MicroDrop software as described previously. ^37^ A plugin for the MicroDrop software was used to control a permanent magnet fitted with a magnetic field lens (described previously^30^) attached to a servomotor positioned beneath the DMF device. The magnetic lens could be “activated” (positioned close to the device, such that the magnetic field can immobilize particles onto the surface), or “deactivated” (positioned away from the device, such that the magnetic field does not affect particles on the device).

### Wicks

Wicks were prepared from Whatman No. 1 filter paper (Whatman International Ltd., Maidstone, UK), Kimwipe absorbent wipes (Kimberly-Clark, Irving, TX), or SureWick G028 glass fiber. Whatman paper wicks were formed from rectangular substrates of three different sizes: 10 mm × 10 mm, 35 mm × 15 mm, or 35 mm × 30 mm. In each Whatman wick, the edge of at least one substrate was inserted between the bottom and top plates of a DMF device. Wicks formed from 10 mm × 10 mm substrates were positioned over a single reservoir electrode; wicks formed from 35 mm × 30 mm or 35 mm × 15 mm were positioned on top of four reservoir electrodes (with the long edge inserted between the plates). Wicks formed from 10 mm × 10 mm substrates were six-ply (*i.e*., a stack of six substrates), while those from 35 mm × 15 mm or 35 mm × 30 mm substrates were two-ply and one-ply, respectively. Kimwipe wicks were formed from triangular (10 mm × 15 mm) and rectangular (30 mm × 110 mm) substrates. Each Kimwipe wick was formed by (i) loading a triangular substrate into a DMF reservoir such that the vertex angle penetrated between the top and bottom plates, and (ii) positioning a rolled up rectangular substrate on top of the triangular substrate. Finally, SureWick wicks were formed from 10 mm × 10 mm substrates. Each SureWick wick was two-ply and was integrated into a DMF device as described for the Whatman wicks, above. The various wick geometries are shown in Fig. S1 in the ESI.†

### P-CLIP general procedure

The P-CLIP procedure is performed in four steps: (I) a volume of sample containing functionalized paramagnetic particles and bound analyte was loaded onto a DMF reservoir electrode opposite a “wicking reservoir” containing an absorbent wick. (II) The magnetic lens beneath the DMF device was activated, and a virtual channel (VC) of sample was formed by activating a linear path of electrodes connecting the sample reservoir to the wicking reservoir. The sample flowed through the VC, driven by adsorption into the wick. (III) The particles accumulated over the magnet. When the majority of sample became absorbed into the wick, electrodes were sequentially turned off starting at the sample reservoir. (IV) After all the sample-supernatant adsorbed into the wick, the magnet was disengaged. A droplet of buffer was dispensed, driven to the particles, and mixed to resuspend them.

### Flow rate characterization

P-CLIP flow rates were characterized using two tests with 75 μL samples of PBS (with no particles). In the first test, the general P-CLIP procedure (steps I–IV) was applied for each of the wicks described above. Each condition was evaluated in triplicate, and the average time required to imbibe the entire sample was observed and recorded. The second test was conducted with SureWick wicks. In each experiment, the mass of the wick was measured, and steps (I–II) of the general P-CLIP procedure were implemented, terminating the experiment by removing the wick at 3, 4, 5, or 6 seconds after forming the VC. Immediately after termination, the mass of the wick was measured again. The pre- and post-experiment difference in mass and a density of 1.0 g cm^−3^ were used to calculate the volume of buffer absorbed and consequently the average flow rate of the VC for each time point.

### Recovery efficiency

Recovery efficiency of the P-CLIP method was determined for magnetic microparticles of three different diameters—2.8 μm (Dynabeads M-270 Streptavidin; Thermo Fisher Scientific, Mississauga, ON), 5 μm (Architect Rubella IgG particles; Abbot Laboratories, Abbot Park, IL), and 10 μm (PureProteome^™^ Albumin Magnetic Beads; EMD Millipore, Etobicoke, ON). Stock suspensions of each type were prepared at densities of 9.00 × 10^7^ to 1.84 × 10^8^ particles per mL. The densities of each suspension were measured by diluting a 2 μL aliquot to 100 μL with PBS and then counting the particles in 10 μL of the diluted suspension using a haemocytometer (in triplicate). 2 μL aliquots of stock suspensions were then diluted into 50, 75 and 100 μL PBS (final volumes) and processed using P-CLIP with SureWick wicks. After the sample had been processed, the top plate of the DMF device was removed, a 100 μL aliquot of fresh PBS was pipetted onto the concentrated particles, and aspirated up and down to collect them. The density of the resuspended sample was then measured by counting in a haemocytometer, as above. The recovery efficiencies for each condition were calculated as the ratio between the number of particles measured pre- and post-P-CLIP processing.

### P-CLIP volumes and samples

For modest sample volumes (≤100 μL) P-CLIP experiments were conducted without modification of the sample reservoir. For large volumes (>100 μL), a wax barrier was formed around the border of the reservoir electrode on the DMF by melting and dispensing beeswax with a “kistka” electric wax pen (Folk Impressions, Franklin, MI). The P-CLIP procedure was performed on the following solutions (all containing 0.1% Tetronic 90R4): (a) a 2 μL aliquot (100 mg mL^−1^) of 2.8 μm dia. particles (DynaBeads® M-270 streptavidin) added to 75 μL of centrifuged pooled human saliva (Lee Biosolutions, Maryland Heights, MO), (b) a 4 μL aliquot (100 mg mL^−1^) of DynaBeads® MyOne^™^ streptavidin 1 μm dia. particles (Thermo Fisher Scientific, Mississauga, ON) added to 100 μL of human urine (BioChemed, Winchester, VA), (c) a 4 μL aliquot (100 mg mL^−1^) of DynaBeads® MyOne^™^ streptavidin 1 μm dia. particles added to 100 μL of bovine serum, and (d) a 4 μL aliquot of 2.8 μm dia. particles (100 mg mL^−1^) (DynaBeads® M-270 streptavidin) added to 300 μL of PBS.

### DMF-ELISA

Most droplet volumes used in the DMF ELISA were 2.4 μL (known as a double-unit droplet), as defined by the area of two DMF driving electrodes and the inter-plate height (180 μm) on inkjet-printed devices. MicroDrop v 2.0 software37 was used to write a series of basic subroutines including ‘move’, ‘dispense’, ‘separate’, ‘resuspend’, ‘mix’, and ‘split’. For move (and other subroutines), the DropBot control system applied electrical potentials between the top plate and sequential driving electrodes of the bottom plate. For dispense, a reservoir electrode and four driving electrodes were actuated for 8 s, after which the two middle electrodes were turned off for 9 s, allowing a double-unit droplet to pinch off. For separate, the magnetic lens was activated under a droplet containing paramagnetic particles while the supernatant was moved away, typically to waste. For resuspend, the magnetic lens was deactivated and a droplet was moved onto the particles and moved over a series of 11 electrodes in a circular pattern for 20 s. For mix, droplets were moved over a series of electrodes in a circular pattern for a defined amount of time. For ‘split’, electrodes on either side of a merged droplet (formed from two double-unit droplets) were actuated while the central electrodes were turned off for 9 s, allowing the fluid to pinch into two double-unit droplets.

The DMF-ELISA was performed in eight steps to evaluate solutions of *Pf*LDH in sample diluent at different concentrations. (1) A double-unit droplet of paramagnetic particle suspension functionalized with capture antibody was dispensed onto the array of driving electrodes and then the particles were separated from the supernatant. (2) A double-unit droplet of sample was dispensed and used to resuspend the particles, which were then mixed for 5 min. (3) The particles were separated from the sample and then washed two times by sequentially dispensing a double-unit of wash buffer, resuspending the particles, and separating the droplet to waste. (4) A doubleunit droplet of conjugated detection antibody was dispensed and used to resuspend the particles, which were then mixed for 5 min. (5) The detection antibody supernatant was separated and removed to waste, and the particles were washed four times in the same fashion as step 3. (6) Separate double-unit droplets of luminol/enhancer and H_2_O_2_ solutions were dispensed, merged, mixed for 30 s, and finally split into two double-unit droplets. (7) One of the double-unit droplets of luminol/H_2_O_2_ substrate was used to resuspend the particles which were then mixed for 7 min. (8) The droplet containing the paramagnetic particles was moved to the detection zone and chemiluminescence was measured by the integrated photomultiplier (PMT) (Hamamatsu H10721-01, Hamamatsu Corp., Bridgewater, NJ). In each measurement, 10 s of signal was collected at 60 Hz, recording the mean of the final 8 s. Typically, the DMF-ELISA was performed on four samples in parallel, with the normalization standard (100 ng mL^−1^ *Pf*LDH) always assayed as one of the four samples.

When combined with P-CLIP, the DMF-ELISA procedure comprised two modified steps, (1a) and (2a), followed by steps (3–8) from the general DMF-ELISA procedure described above. (1a) A 2.4 μL aliquot of paramagnetic particle suspension (6.7 × 10^8^ particles per mL) was added to 75 μL of sample in a microcentrifuge tube. The sample and particles were rotated at room temperature for 3 h. (2a) The four-step P-CLIP procedure described above (I–IV) with SureWick wicks was used to load the particles into the device, resuspending them into a double-unit droplet (2.4 μL) of buffer in step (IV). The normalization standard (100 ng mL^−1^ *Pf*LDH) was always run using the conventional DMF-ELISA (without PCLIP).

### Statistical analysis

Prism 6 by GraphPad was used to perform statistical analysis. Two-way ANOVA with a Tukey's *post hoc* test with *α* = 0.05 was used to determine significance amongst recovery efficiencies. Each DMF-ELISA measurement signal was divided by the signal from the concurrently measured normalization standard (100 ng mL^−1^) and multiplied by 1.70 × 10^−9^ A, plotted as function of concentration, and fit by linear regression. The limit of detection (LOD) and limit of quantification (LOQ) were defined as the concentrations corresponding to the mean signal of the blank plus three or ten standard deviations of the blank, respectively (with *y*-axis values: *y*_lod_ = *y*_blank_ + 3*σ*_blank_; *y*_loq_ = *y*_blank_ + 10*σ*_blank_). A Student's *t*-test with α = 0.05 was used to determine significance between traditional and P-CLIP DMF ELISAs.

## results and discussion

### Development, optimization, and characterization of P-CLIP

**Fig. 1:**
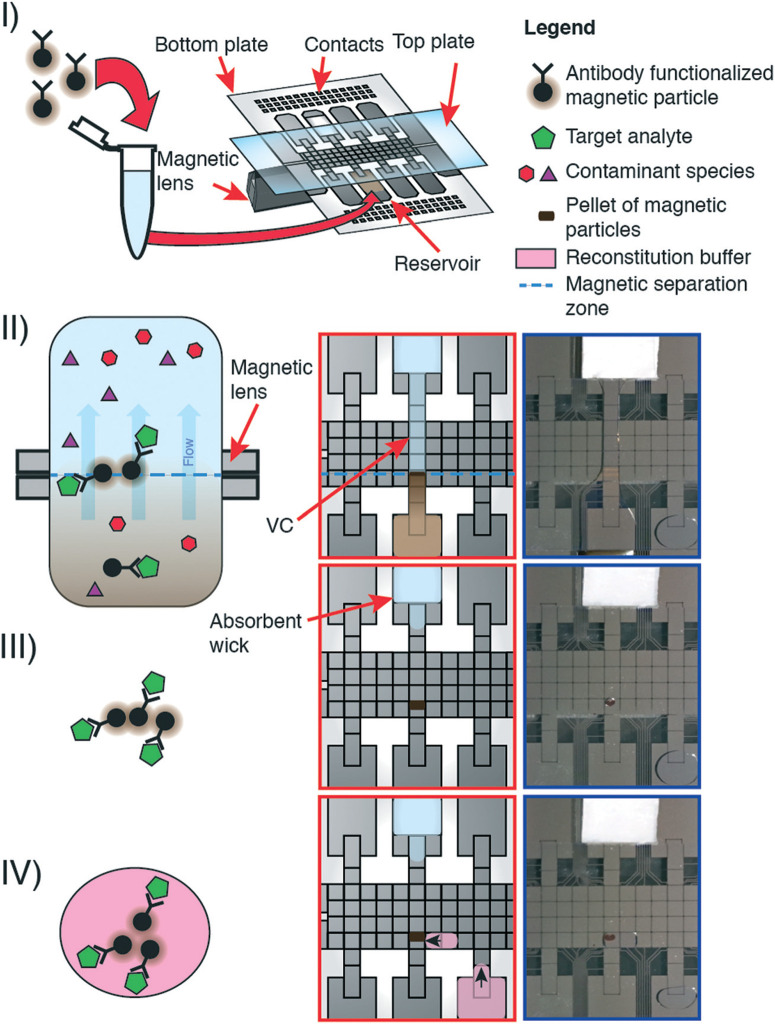
Pre-concentration by liquid intake by paper (P-CLIP). Cartoon (left) illustrating the P-CLIP method at the microscale; cartoon (centre) and photographs (right) illustrating the corresponding steps of P-CLIP on DMF. I) magnetic beads functionalized with antibodies that are specific to the target analyte are incubated with the sample off-chip in a microcentrifuge tube. After incubation, the whole sample is loaded into the reservoir of a DMF device comprising a top and bottom plate. A moveable magnetic lens is positioned beneath the device. II) A virtual channel (VC) is formed by DMF electrode actuation connecting the sample reservoir to an absorbent wick. The magnetic particles are trapped within the magnetic field at the magnetic separation zone while the supernatant continues to flow. III) Supernatant is removed from the magnetic particles. IV) The magnetic particles are reconstituted in a smaller volume of reconstitution buffer.

We developed pre-concentration by liquid intake by paper (PCLIP) in response to the challenge of working with analytes present in low concentrations in digital microfluidics. As shown in Fig. 1, P-CLIP is implemented in four steps, in which a sample is (I) exposed to antibody-functionalized magnetic particles, such that antigens in the sample become bound, (II) the sample/particle suspension is loaded into a DMF device and electrodes are actuated to form a virtual channel (VC), (III) the particles become immobilized over the magnet as the fluid is absorbed into an absorbent wick, and (IV) the particles are collected into a fresh droplet, where the maximum pre-concentration factor is determined by the ratio of the original volume to the collection-droplet volume.

The most critical aspect of P-CLIP is the formation of a VC, a technique in which a barrier-free “channel” of fluid is formed by simultaneously applying voltages to a series of adjacent electrodes connecting a source liquid sample to an absorbent sink (which draws out the liquid sample). The VC allows large volumes of sample to be continuously and efficiently processed on a DMF device in a single step; the alternative of processing a large number of small-volume droplets in series would be unwieldy and slow. The clever idea of creating VCs on DMF has been described before, primarily as a means of generating reconfigurable fluid-flow paths.^38,39^ There are two reports using VCs on DMF as a tool to handle paramagnetic particles. In the first example (described in a Ph.D. thesis^40^), a VC driven by a syringe pump was used to flow buffer over a pellet of immobilized paramagnetic microparticles to wash them. In the second example,^41^ a VC was formed to connect a first droplet containing magnetic microparticles to second droplet; application of a magnetic field pulled the magnetic particles along the VC, sequestering them in the second droplet. These methods were inspirational in our development of P-CLIP, but note that neither of them used VCs for pre-concentration, and only the former could conceivably be used for this purpose. Unlike the former, in P-CLIP, fluid-flow is driven by a passive capillaryaction pump^42,43^ (at the sink), which maintains the “no moving parts” philosophy of digital microfluidics.

Several P-CLIP wick materials and configurations (Table 1; Fig. S1†) were tested with the goal of determining a facile setup that removed supernatant rapidly and reproducibly. Whatman No. 1 paper was the first material evaluated, motivated by its popularity in paper microfluidics.^44–46^ Initially, a large wick (35 mm × 30 mm) that extended the length of four reservoir-electrodes was evaluated and found to be slow – 120 s to imbibe the test volume, 75 μL. This led us to double the cross-sectional area of the wick material by stacking two substrates on top of each other, informed by Darcy's law^47^

**Table 1:** P-CLIP imbibition times for different configurations of absorbent wicks

Material	Whatman No.1	Whatman No.1	Whatman No.1	SureWick	Kimwipe
Geometry	One-ply 35mm × 30mm substrates	Two-ply 35mm × 15mm substrates	Six-ply 10mm × 10mm substrates	Two-ply 10mm × 10mm substrates	One 10 mm × 15 mm triangle substrate coupled to a 30 mm × 110 mm roll
Approximate time required to imbibe 75 μL PBS	120s	60s	60s	7s	10s

$$Q\;=\;\frac{k\;A\;\left(P_{b}\;-\;P_{a}\right)}{\mu L}$$( 1 )

where volumetric fluid flow rate *Q* is proportional to the wick's permeability *κ*, cross-sectional area A, and pressure drop *p*_b_ − *p*_a_, and inversely proportional to the fluid viscosity *μ* and the length of the pressure drop *L*. Darcy's law indicates that doubling *A* by stacking two substrates (while maintaining the total amount of material) should halve the imbibition time, which matched experimental observation: 60 s to imbibe the test volume. This gain was appreciated, but the size of the first two wicks tested (which covered half of the device) was impractical. The next configuration tested increased *A* again (to a stack of six substrates), but the total amount of material was reduced such that the wick matched the footprint of a single reservoir electrode (10 mm × 10 mm). This combination proved to have nearly identical performance as the second configuration: 60 s to imbibe the test volume. In an attempt to maintain the same wick-footprint but to increase the imbibition velocity, we switched to the highly absorbent material SureWick (altering *κ* and *p*_b_ − *p*_a_). It was found that two-ply 10 mm × 10 mm SureWick wicks offered very rapid imbibition (7 s) and the desired footprint. As a reference, we also tested triangles cut from Kimwipe tissues, which we have used previously to wick away unwanted waste on a DMF device^28^ or as a wick when P-CLIP was applied for proteomic sample concentration for mass spectrometry. ^27^ These wicks also had fast imbibition times (10 s) but were tedious to cut and assemble. Thus, two-ply 10 mm × 10 mm wicks formed from SureWick material were used for all subsequent experiments.

**Fig.2:**
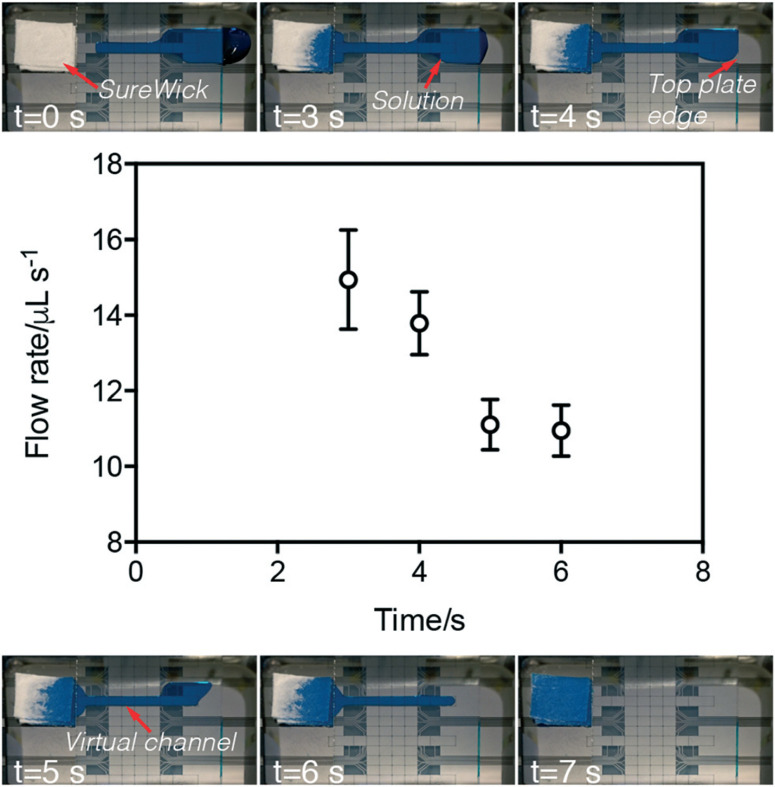
Analysis of flow rate in P-CLIP virtual channels. Top and bottom: Video frames depicting six time-points during a P-CLIP imbibition of 75 μL of PBS (with blue dye added to aid in visualization). Middle: Plot of flow rate measured as a function of time for the process (open circles). Error bars represent ± 1 std. dev. (*n* = 3).

Flow velocities within VCs were characterized by measuring the difference in mass of the wick before and after P-CLIP was initiated and terminated (Fig. 2). As shown, over the course of VC formation and processing, the average flow rate begins high and then decreases over time, with an inflection observed between the two regimes. We propose two possible hypotheses that may explain this phenomenon. One hypothesis is that the inflection point coincides with the source-reservoir transitioning from extending outside of the two plates to being completely sandwiched by the two plates. In this case, the LaPlace pressure exerted by external reservoir may account for the high flow rate observed initially; as the source-fluid becomes sandwiched between the top and bottom plates, this pressure is reduced. As a second hypothesis, we note that fluid flow within a channel driven by a passive paper pump can be described as a function of the change in the radius of wetting the paper pump.43 With this in mind, we hypothesize that the radius of wetting initially increases rapidly as the VC is imbibed, but eventually reaches the boundary of the wick and then slows. More work is needed to characterize the two potential hypotheses (or other explanations); regardless, the phenomenon was found to be useful for different volumes, solutions, and applications, as described below.

**Fig.3:**
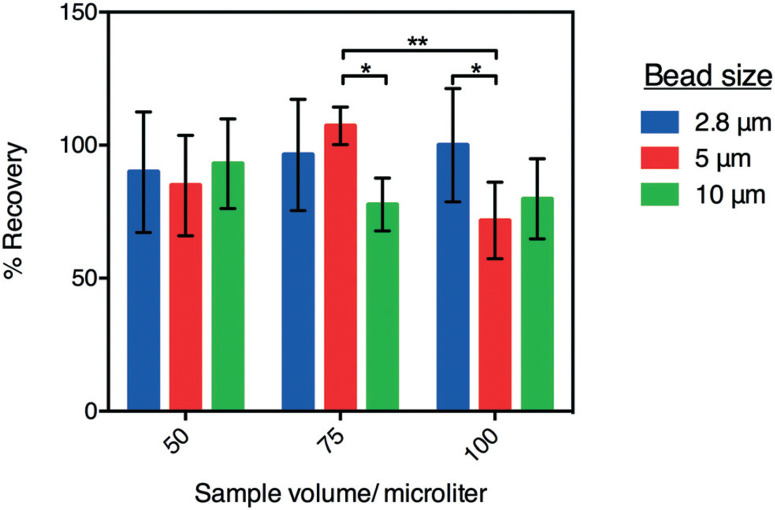
P-CLIP particle recovery efficiency. Plot of the percentage of particles counted before and after P-CLIP for 2.8 μm – (blue) 5 μm – (red) and 10 μm – (green) diameter magnetic particles from 50, 75, or 100 μL volumes of PBS. Error bars represent ± 1 relative std. dev. (*n* =3). *P ≤ 0.05, **P ≤ 0.01.

As a final characterization of P-CLIP, we tested the recovery efficiency of magnetic particles of different sizes, collected from different sample volumes. In these experiments, the recovery rate was evaluated for 2.8, 5, and 10 μm-diameter paramagnetic particles collected from 50, 75, and 100 μL sample volumes of PBS (Fig. 3) using a haemocytometer. As shown, for particles recovered from 50 μL, recovery efficiencies were within one standard deviation of 100% and there was no significant difference between different particle diameters (*P* = 0.0560). Further, 2.8 μm dia. particles were recovered at the same high efficiency from all volumes tested, while larger particles were recovered progressively less efficiently from larger volumes, as evidenced by the statistically significant decrease in recoveries amongst larger particles in larger volumes. A possible explanation for this phenomenon is that larger particles have greater momentum and therefore pass more readily through the magnetic capture zone. To overcome this, flow rates could be decreased or alternatively the magnetic field-strength (in this case a remnant field strength of 1.32 T focused to exert ∼500 μN force on the particles30) could be increased. In the current study, 2.8 μm dia. particles were used for the applications tested (described below).

### Application of P-CLIP to complex samples

**Fig. 4:**
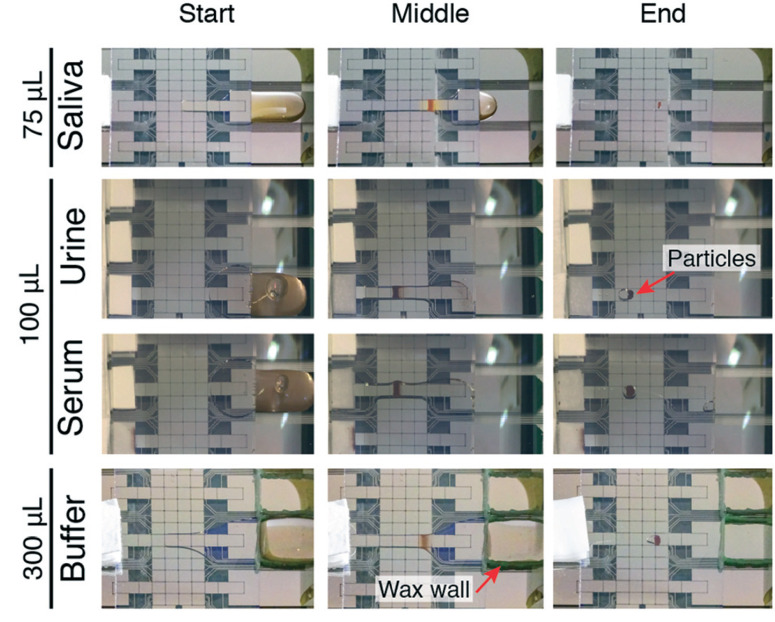
P-CLIP fluids and volumes. Photographs of the start, middle, and end of particle concentration procedures applied to saliva, urine, serum, and buffer samples. Top: 4 μL of 2.8 μm dia. particles are concentrated from 75 μL of human saliva. Second from top: 4 μL of 1 μm dia. particles are concentrated from 100 μL of human urine. Second from bottom: 4 μL of 1 μm dia. particles are concentrated from 100 μL bovine serum. Bottom: 4 μL of 2.8 μm dia. particles are concentrated from 300 μL of PBS. The latter required the use of a large sample reservoir bounded in a wax wall.

We developed P-CLIP to assist with detection of disease biomarkers present at low concentrations in biological fluids. With this in mind, we tested the applicability of P-CLIP to recover paramagnetic particles from saliva, urine and serum (Fig. 4) – each of the fluids proved to work well, with no obvious differences relative to buffer. In addition, while 75–100 μL may be considered “large” by microfluidic standards, we also tested the capability of the P-CLIP procedure with an even larger sample volume, 300 μL. The only other method to report the use of a comparable volume coupled to DMF33 relied on an external peristaltic pump; P-CLIP replaces the pump (and associated tubing) with a passive sink with no moving parts.

**Fig. 5:**
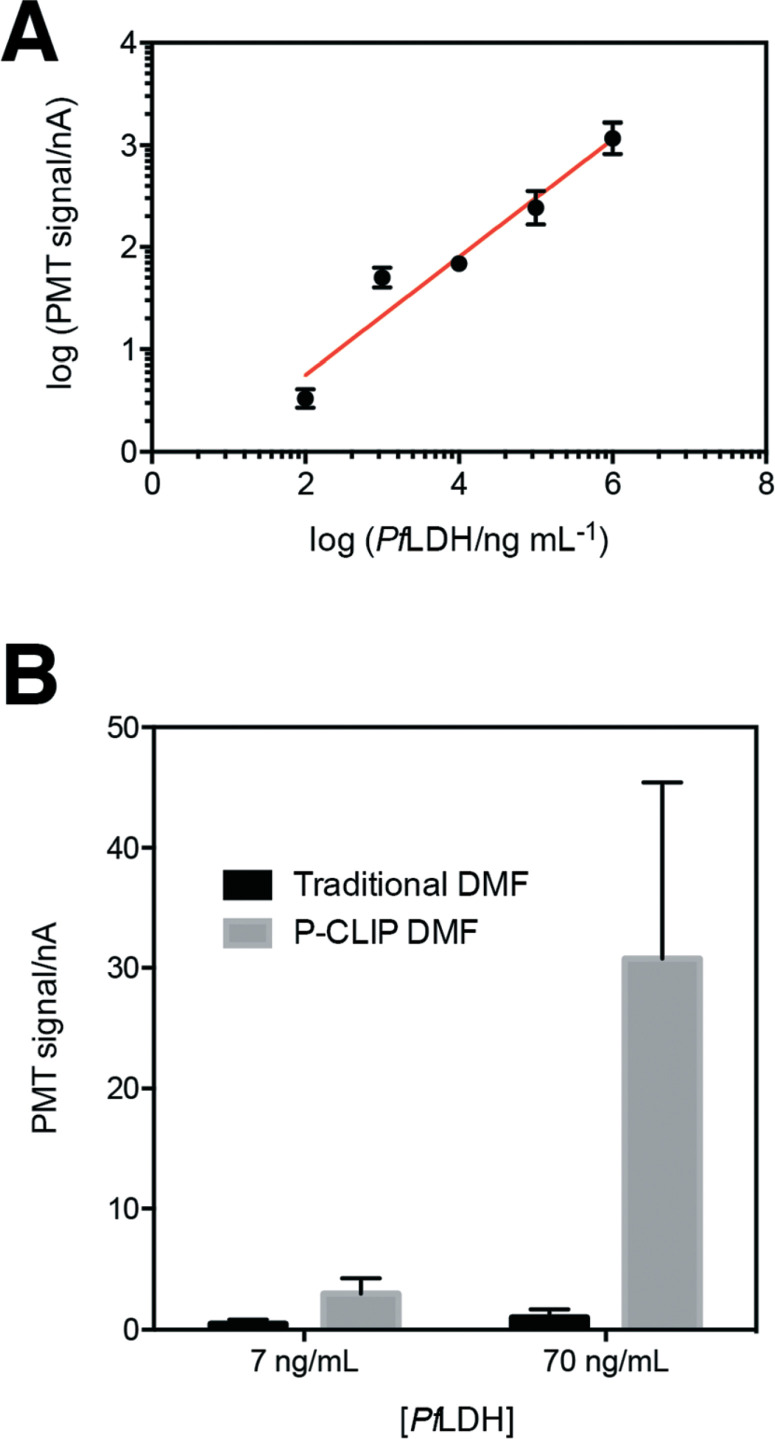
DMF immunoassay for PfLDH. (A) Standard curve for *Pf*LDH as determined by traditional DMF-ELISA. Error bars represent ± 1 std. dev. (*n* = 3). (B) Comparison of mean signals obtained by traditional DMFELISA (black) and P-CLIP modified DMF-ELISA (grey) for concentrations below the limit of detection (7 ng mL^−1^) and limit of quantitation (70 ng mL^−1^) of the traditional DMF-ELISA. Error bars ± 1 std. dev. (*n* = 3).

Having established P-CLIP's compatibility with various biological samples, we applied P-CLIP to a chemiluminescent DMFELISA to investigate themethod's impact on assay performance. We developed a DMF-ELISA for *Pf*LDH and prepared a standard curve (Fig. 5A) using recombinant *Pf*LDH in 2.4 μL droplets at concentrations ranging from 0–1.0 × 10^6^ ng mL^−1^. As far as we are aware, this represents the first report of a DMF method designed to test for an antigen specific for malaria or any other non-viral infectious disease. Least-squares analysis was used to determine a line of best fit [logĲsignal_PMT_) = 0.5776 logĲ*Pf*LDH) − 0.4074; *R*^2^ = 0.9293], with a limit of detection (LOD) and limit of quantitation (LOQ) of 8.8 ng mL^−1^ and 83.9 ng mL^−1^, respectively. Unfortunately, these values are too high to be used for detecting the presence of malaria, as *Pf*LDH is found in the sera of malaria-infected patients at the ∼1–15 ngmL^−1^ range.^35^ Thus, P-CLIP was used to pre-concentrate the analyte prior to analysis. 75 μL samples with concentrations just below the LOD and LOQ (7 ng mL^−1^ and 70 ng mL^−1^) were evaluated using the P-CLIP modified DMF procedure (Fig. 5B). By applying the pre-concentration step, signals improved 6.6-fold for 7 ng mL^−1^ and 30.9-fold for 70 ng mL−1 relative to standard measurements generated using 2.4 μL samples with no pre-concentration. These improvements were significant relative to the standard measurements (*P* = 0.0288 for 7 ngmL^−1^ and *P* = 0.0244 for 70 ng mL^−1^), but they did come with increased error (42–47% CV), likely caused by irreproducible particle loss during the incubation step. We propose that in the future, both the incubation time and error may be reduced by coupling a high-capacity reservoir with the DMF device and using more efficient mixing by magnetic actuation. ^48^ Further, the experiment presented here was designed as a head-to-head comparison, keeping the total number of particles the same between conventional and P-CLIP DMF immunoassays. It is likely that by increasing the number of particles used with P-CLIP, incubationtimes could be decreased while still allowing large samples to be processed by DMF.

## Conclusions

We developed P-CLIP in response to the challenge of interfacing large sample volumes containing low-abundance analytes with DMF for sample workup and analysis. This simple method capitalizes on the ability to generate defined paths for fluid flow (virtual channels) using DMF electrodes and wicking forces generated by absorbent materials. We characterized several aspects of this new pre-concentration method and demonstrate its suitability to a range of sample types and particle sizes. We demonstrated P-CLIP's utility in a DMF ELISA for the malaria biomarker *Pf*LDH and propose that P-CLIP will be a useful tool for concentrating low-abundance analytes for a wide range of applications.
