# Supplementary material for: Pre-concentration by liquid intake by paper (P-CLIP): a new technique for large volumes and digital microfluidics
Source: Lab Chip. 2017 Jun 12;17(13):2272–80. doi: 10.1039/c7lc00440k (PMC7734381; doi:10.1039/c7lc00440k)
Supplement: Supplementary file 1 [file LC-17-2272-s001.pdf]

Electronic Supplementary Material (ESI) for Lab on a Chip.
This journal is © The Royal Society of Chemistry 2017

Supplementary Information for

Pre-Concentration by Liquid Intake by Paper (P-CLIP): A New Technique for Large Volumes and Digital Microfluidics

Darius G. Rackus[1,2], Richard P.S. de Campos,[1,2], Calvin Chan[1], Maria M. Karcz[1,2], Brendon Seale[1], Tanya Narahari[1,2], Christopher Dixon[1], M. Dean Chamberlain[1,2], and Aaron R. Wheeler[1,2,3†]

[1] Department of Chemistry, University of Toronto, 80 St George St., Toronto, ON, M5S 3H6, Canada

[2] Donnelly Centre for Cellular and Biomolecular Research, 160 College St., Toronto, ON, M5S 3E1, Canada

[3] Institute of Biomaterials and Biomedical Engineering, University of Toronto, 164 College St., Toronto, ON, M5S 3G9, Canada

[†] Corresponding Author
email: aaron.wheeler@utoronto.ca
tel: (416) 946 3864
fax: (416) 946 3865

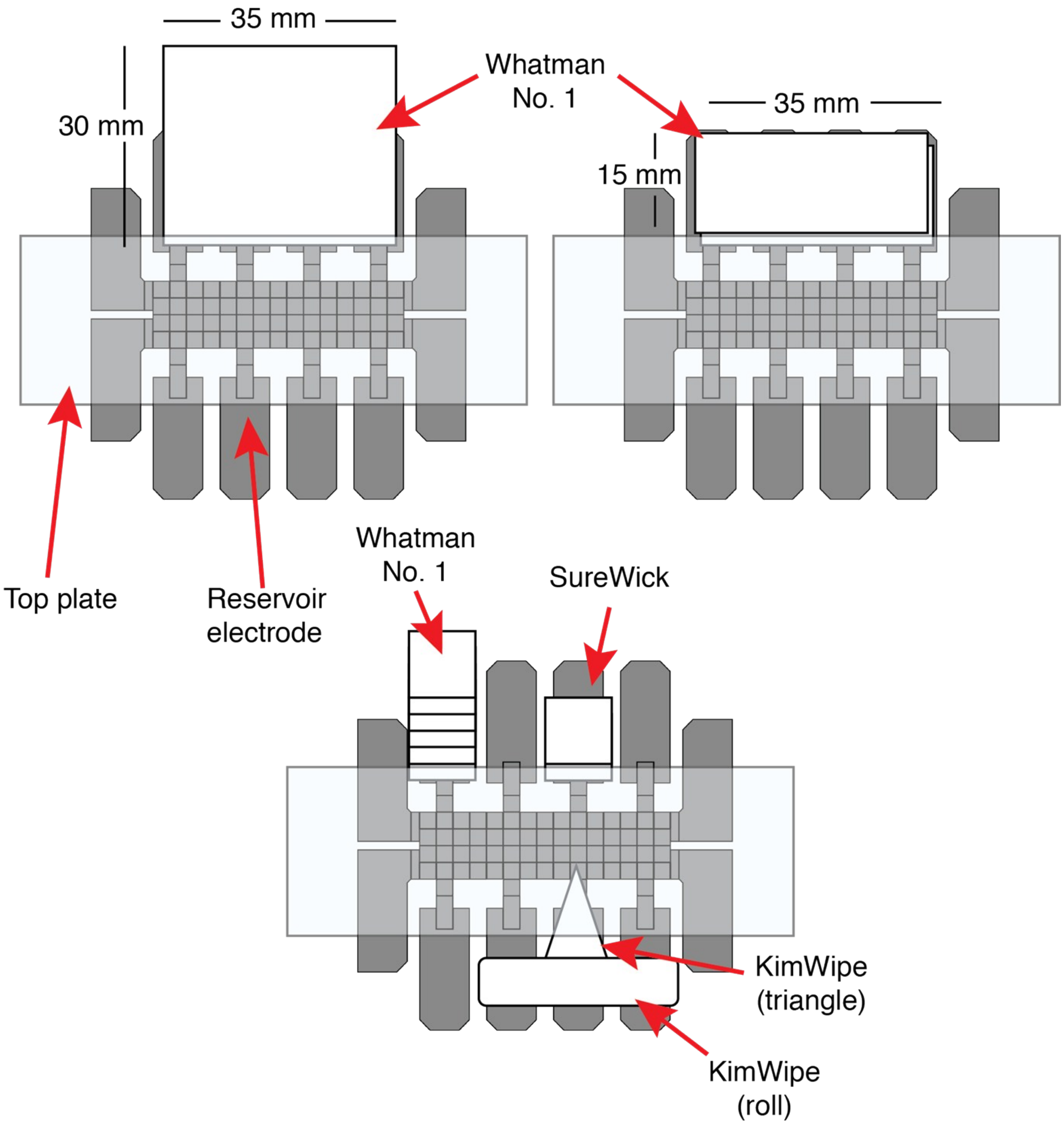

Figure S1. Cartoon illustrating the various wick formats that were tested for P-CLIP performance. The large Whatman No. 1 wicks (top) were inserted over a group of reservoir electrodes between the top and bottom plates of the DMF device by the long edge. The smaller wicks (bottom) comprised multiple layers and the bottom wick was inserted between the top and bottom plates of the DMF device.
